# The effects of centering pregnancy on maternal and fetal outcomes in northern Nigeria; a prospective cohort analysis

**DOI:** 10.1186/s12884-018-1805-2

**Published:** 2018-05-11

**Authors:** George I. Eluwa, Sylvia B. Adebajo, Kwasi Torpey, Oladapo Shittu, Shittu Abdu-Aguye, Daniel Pearlman, Umma Bawa, Aira Olorukooba, Hadiza Khamofu, Robert Chiegli

**Affiliations:** 1 Population Council, No. 16, Mafemi Crescent, Utako, Abuja, Nigeria; 2 University of Ghana College of Health Sciences, Accra, Ghana; 3 Department of Obstetrics & Gynaecology, Ahmadu Bello University Teaching Hospital, Zaria, Kaduna Nigeria; 4 Department of Paediatrics, Ahmadu Bello University Teaching Hospital, Zaria, Kaduna Nigeria; 5 Johns Hopkins Center for Communication Programs, Abuja, Nigeria; 6 Bixby School of Public Health, University of California, Berkeley, USA

## Abstract

**Background:**

Maternal and infant mortality remains high in Nigeria primarily due to low use of skilled birth attendants. Huge disparities exist between southern and northen Nigeria on use of skilled birth attendants with south significantly higher than the north. We assessed the effect of centering pregnancy group (CPG) antenatal care on the uptake of antenatal care (ANC), facility delivery and immunization rates for infants in Kano state.

**Methods:**

Between December 2012 and May 2014, pregnant women with similar sociodemographics and obstetric history were enrolled into intervention (CPG) and control groups and followed up prospectively. Chi-square tests were conducted to compare the differences between the intervention and the control groups with respect to background characteristics and intervention outcomes. Logistic regression was used to measure the associations between CPG and uptake of services for mother-baby pairs in care.

**Results:**

A total of 517 (260 in the control group and 257 in the CPG) pregnant women enrolled and participated in the study. Thirty-six percent of women in the control group attended ANC at least once in 2nd and 3nd trimester compared to 49% of respondents in the CPG (*p* < 0.01). Health facility delivery was higher among CPG (13% vs. 8%; *p* < 0.01). When controlled for age, number of previous pregnancies, number of term deliveries, number of children alive and occupation of respondent or their spouses, respondents who participated in the CPGs compared to those who did not, were more likely to attend at least one antenatal care (ANC) session in the third trimester [adjusted risk ratio (ARR):1.52; 95% CI:1.36–1.69], more likely to immunize their babies at six weeks [ARR: 2.23; 95% CI: 1.16–4.29] and fourteen weeks [ARR: 3.46; 95% CI: 1.19–10.01] and more likely to use health services [ARR: 1.50; 95% CI: 1.06–2.13].

**Conclusion:**

Centering or group pregnancy showed a positive effect on the use of antenatal services, facility delivery and postnatal services and thus is a promising intervention to increase uptake of maternal health care services in northern Nigeria. The low facility delivery remains a cause for alarm and requires further investigation to improve facility delivery in northern Nigeria.

## Background

“*Every Woman Every Child*” is the World Health Organization’s global strategy for ending all preventable deaths of women, children and adolescents within a generation and ensuring their well-being [1]. The ability to implement innovative and evidence-based antenatal care practices is critical to the success of Every Woman, Every Child. Within the continuum of reproductive health care, antenatal care (ANC) provides a platform for important health-care interventions, including health promotion, screening and diagnosis, and disease prevention. It has been established that by implementing timely, appropriate and low-cost evidence-based ANC practices we can save lives [1] and ensure that women deliver with the assistance of skilled attendants [2, 3].

A review of World Health Statistics in 2015 shows that ANC coverage is indirectly correlated with maternal mortality ratio (MMR) worldwide, indicating that countries with low ANC coverage had high MMR. Compared to Ukraine and The United Arab Emirates with ANC coverage of 99 and 100% respectively, MMR was 24 and 6 per 100,000, while for Chad and Nigeria with ANC coverage of 43 and 61% respectively, MMR was 980 and 560 per 100,000 in 2013 [2, 4–7]. ANC enhances early screening, identification and management of threatening conditions that could lead to poor maternal and fetal outcomes. Access to skilled health providers in the continuum of ANC reduces incidence of anaemia, hypertension, ectopic pregnancy, obstructed labour, eclampsia, excessive bleeding, premature labour and delivery [2, 5, 8–12].

In Nigeria, utilization of maternal healthcare services has remained poor. A national survey in 2013 showed that less than two-thirds of women who had a live birth in the five years preceding the survey received ANC from a skilled provider and only about half reported making four or more ANC visits during pregnancy [8, 13, 14]. Furthermore, less than two-fifths of deliveries within the five years were delivered in health facilities and assisted by skilled providers [8]. Across Nigeria, there are wide variations in utilization of maternal health services with southern Nigeria reporting higher utilization than northern Nigeria. While use of ANC services was 91, 90 and 73% in the south east, south west and south south geo-political zones respectively, it was 67, 49 and 41% for the north central, north west and north eastern geo-political zones respectively. This variation also persists in places of delivery. Delivery in a health facility was 78, 75 and 50% for south east, south west and south south geo-political zones respectively while it was 46, 20 and 12% for the north central, north west and north eastern geo-political zones respectively [14]. There is also disparity in ANC coverage between urban and rural settings and this has been attributed to several factors including inequities in the number of accessible health facilities [10, 15]. For infant immunization, north-south disparity persists with immunization coverage of 10, 14 and 27% for north west, north east and north central regions respectively and 52% for south east and south south regions and 41% for south west region. In Nigeria, the urban bias in public health expenditure, inadequate financing coupled with difficulties in attracting and retaining health workers in rural areas have contributed significantly to the government’s inability to create an accessible community health care system [8, 15].

Centering Pregnancy (CP), a group prenatal care model, is a promising innovation which challenges the standard model of one-on-one counselling of prenatal care [16–19]. Centering Pregnancy replaces the individual prenatal care visit with a group model for obstetrically low-risk women, and this model provides substantially more health promotion content than the traditional one-on-one prenatal care model. Elements unique to group care include group peer support and self-management training and activities [16, 19]. Group delivery of health care holds promise as a model that can increase health promotion content and social support as well as lead to behavior change [16, 20]. Discussing more health promotion topics during pregnancy has been associated with healthier behaviours during pregnancy and more positive attitudes during pregnancy [16, 21]. In addition, CP model aligns with “focused or goal oriented ANC” which include the following essential elements- recognition and management of pregnancy related complications; screening for conditions and diseases; health promotion (use of immunization, hematinics and insecticide treated nets); and counseling and support to the woman and her family to develop healthy home behaviours [22].

This paper describes a centering pregnancy intervention and assessed its effects on utilization of healthcare services for both mothers and their babies in Kano state, Nigeria. Specifically, we assessed the number of ANC visits, delivery in the health facility for the mothers and immunization completion rates for their babies. Such information is useful for informing, planning, and providing targeted programming for maternal and child health services in states with poor maternal and child healthcare utilization rates in Nigeria.

## Methods

### Setting

Kano State is situated in the North-West of Nigeria and administratively divided into 44 Local Government Areas (LGAs). It is the most populous northern state with a total population of 9.4 million people of which 4,627,556 (48.3%) are female [23, 24]. Women of child-bearing age (15–49 years) account for about one-fifth of the total population, while the number of pregnant women (5%) in the state translates to about 478,280.

### Program description

The study was conducted in Kura Local Government Area (LGA) of Kano State, Nigeria, a largely rural community between December 2012 and May 2014. Kura LGA had a projected population of 175,200 with 38,544 women of childbearing age and 8760 pregnant women. Four intervention community clusters in Kura LGA were selected for the establishment of the centering pregnancy groups (CPGs). The intervention community clusters were selected based on having a health facility where deliveries were taken to ensure that requisite staff (nurse/midwife) and infrastructure for delivery was available. Each community cluster had a complement of four CPGs – for primigravida, multigravida, grand multigravida and postnatal care. The purpose of segregating pregnant women into groups defined by age bands and number of pregnancies was to facilitate peer-to-peer interaction during the CPG sessions by eliminating the cultural expectations of deference to older people, which would be a significant barrier to open interactions. Thus, a total of 16 CPGs were created as intervention groups.

The CPG curriculum was based on a community-validated facilitative approach, which incorporates locally-rooted cultural concepts, language and practice. The CPG curriculum was adapted from a group pregnancy care model by the Population and Reproductive Health Initiative (PRHI) of Ahmadu Bello University, Zaria, Kaduna state. The CPG curriculum is divided into 11 modules of which eight modules are focused on antenatal care and three modules on postnatal care. During the CPG facilitated sessions, discussions on pregnancy, childbirth and newborn care related topics were guided by a CPG curriculum and basic clinical antenatal care was provided. Topics discussed included knowing your body, common discomforts in pregnancy, nutrition, hygiene, danger signs, birth preparedness, safe delivery, breastfeeding and baby care. In addition, malaria, HIV/AIDS and family planning were discussed. The CP model used in this intervention differed from the standard group ANC approach used at most Nigerian health facilities in the following key respects: (i) an educational format is followed that uses a facilitative leadership style with didactic discussion format; (ii) each session has an overall plan; (iii) attention is given to core content although emphasis may vary; (iv) there is stability of group leadership and the composition of the group is stable, but not rigid; (v) participants are involved in self-care activities and opportunities for socialization are provided and there is ongoing evaluation of outcomes.

Each CPG was facilitated by a team of Community Health Extension Workers (CHEWs). In Nigeria, CHEWs are trained to provide education on pregnancy, manage ANC, recognize signs of labour complications and coordinate referrals for complicated pregnancy but do not undertake labour and delivery services. The CHEWs were trained for three days on the CPG curriculum and facilitation skills before commencement of the intervention. They also had five one-day refresher trainings during the intervention. The CHEW teams were trained and supervised by Obstetricians and Pediatricians from PRHI. Each team consisted of three CHEWs with one acting as the team leader. In addition, each session had a facilitator and a co- facilitator who had different roles: the facilitator introduced the topic for the session, the women facilitated the discussion; and the co- facilitators noted the group dynamics and contributed whenever an important issue in the module was left out or inadequately addressed. The women in each CPG were approximately around the same gestational age hence, the group sessions were scheduled based on the traditionally practiced antenatal care follow-up pattern in Nigeria: monthly visits till 28 weeks of gestation; fortnightly till 36 weeks; and weekly till delivery. After delivery CPG members were required to attend postnatal CPG sessions at 2, 4, 6, 10 and 14 weeks post-delivery in addition to home visits conducted by the CHEWs. The CPG sessions were held at the primary health clinic or center located in the community and each CPG session lasted about 3–4 h. To limit any selection bias, other health care facilities that conduct deliveries within the community clusters were identified. Pregnant women who received regular antenatal and postnatal care at these facilities were identified and recruited as the control group with similar stratification as those in the intervention group. Standard antenatal and postnatal care at health facilities in the LGA had the similar follow-up patterns as described above for the CPGs.

### Study design and sampling

Due to the stratification of pregnant women enrolled in the CPGs by parity, along with the limitation in the number of study sites to work in, randomization at the individual or community level was not possible. Rather, a quasi-experimental non-equivalent groups design was used to to select participants while enabling assignment of study participants by facility catchment to pre-defined study arms.

Using the formula for comparing two proportions, a total sample size of 268 per study arm was required using an ANC utilization rate of 50%, a design effect of 1.5, attrition rate of 20% and level of precision of 0.05 to detect a 15% difference between the intervention group and control group. In anticipation of possible early dropouts and early deliveries (the gestational age at enrollment was based on client self-reports), a total of 587 clients were enrolled into the study.

### Selection of study participants

To be eligible for participation within both intervention and control arms of the study, pregnant women had to be 15–49 years of age, in the second trimester of a normal pregnancy with a single fetus, resident in the political ward in which the CPG was being established and registered at the health facility. Pregnant women were ineligible to participate if they had a pregnancy with complications, such as vaginal bleeding, premature contractions or if clinical assessment suggested that they may require more specialized care. Pregnant women ineligible due to medical exclusion were immediately referred to an appropriate level of care by study clinicians based on existing State Ministry of Health (SMOH) referral protocols. Ethical approval was granted by FHI 360’s Review Board, U.S.A and the National Health Research Ethics Committee (NHREC), Nigeria.

### Data collection and management

CPG facilitators and staff responsible for running ANCs obtained the informed consent of all pregnant women who opted and were eligible to participate in the study. Following this, a questionnaire to capture sociodemographic data was administered to each pregnant woman at enrollment in their local language (Hausa). Antenatal and postnatal client visit information and clinical updates were documented on antenatal and postnatal cards which were held by the study participants, and on antenatal registers which were kept at the health facility. Data from these records were captured electronically on Open Data Kit (ODK) platform installed on android smart phones at each site and stored centrally in a secure server.

### Data analysis

Data were summarized with frequencies and percentages; the quantitative numeric variables such as age, number of deliveries, number of previous pregnancies and number of deliveries were transformed to categorical variables. Given the low utilization of healthcare services in northern Nigeria, we developed a composite variable called “critical uptake of healthcare”, defined as attending ANC in the 3rd trimester and early postnatal care session (within 2 weeks after delivery) or immunization at birth. Chi-square tests were conducted to compare the differences between the intervention and the control groups with respect to each background characteristic and intervention outcome indicator. Bivariate logistic regression analyses were used to test associations between CP and uptake of services for mother-baby pairs in care. Variables significant at *p* < 0.2 were considered for inclusion in multivariate Log-binomial regression models. The models were controlled for age, number of previous pregnancies and number of deliveries, respondent’s occupation and partner’s occupation. The educational status of pregnant women participating in the study was not controlled for in the multivariable model because information was not collected on this variable. However, the study was carried out in rural communities where married women had limited access to formal education beyond primary school level. Statistical analysis was done using STATA 12 software.

## Results

### Background characteristics

**Fig. 1:**
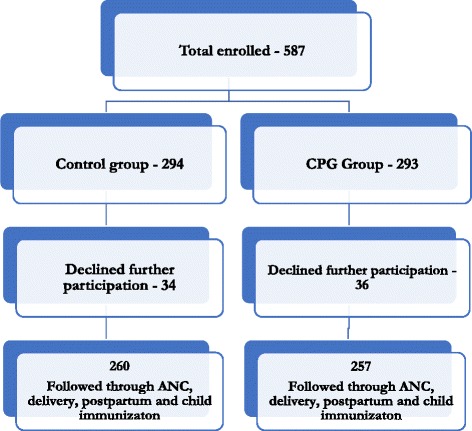
Flow diagram of enrollment and follow-up of pregnant women

Table 1 presents data on background characteristics of the study population. A total of 517 (260 in the control group and 257 in the intervention group) pregnant women who enrolled and participated in the study between December 2012 and May 2014 (Fig. 1). Most respondents were > 25 years in the control (54%) and intervention group (51%; *p* = 0.212). Similar proportion of women in the control (44%) and intervention group (43%; *p* = 0.306) had ≥4 previous pregnancies and had had ≥4 term deliveries (42% vs. 41%; *p* = 0.285). In the control group, a higher proportion of women (41%) had between 1 and 3 children alive while those in the intervention group had ≥4 children alive (35%; *p* = 0.103).

**Table 1:** Background characteristics of study participants

	Control group *N* = 260	Intervention group *N* = 257	Total *N* = 517	
	% (*n*)	% (*n*)	% (*n*)	*p-value
Age (years)				
15–19	16.2(42)	22.2(57)	19.2(99)	0.212
20–24	30.0(78)	26.9(69)	28.4(147)	
≥ 25	53.9(140)	51.0(131)	52.4(271)	
Number of previous pregnancies				
0	23.1(60)	28.8(74)	25.9(134)	0.306
1–3	32.7(85)	28.8(74)	30.8(159)	
≥ 4	44.2(115)	42.4(109)	43.3(224)	
Number of term deliveries				
0	23.1(60)	28.8(74)	25.9(134)	0.285
1–3	35.4(92)	30.7(79)	33.1(171)	
≥ 4	41.5(108)	40.5(104)	41.0(212)	
Number of owned children still alive				
0	25.0(65)	31.5(81)	28.2(146)	0.103
1–3	41.9(109)	33.5(86)	37.7(195)	
≥ 4	33.1(86)	35.0(90)	34.0(176)	
Participants’ job				
Full housewives	91.5(238)	79.4(204)	85.5(442)	0.001
Working (Business, Trade, Artisan or Salary earners)	8.5(22)	20.66(53)	14.5(75)	
Partners’ jobs				
Farming	75.4(196)	37.4(96)	56.5(292)	0.001
Non-farm jobs	24.6(64)	62.7(161)	43.5(255)	

* Chi square, significance observed if *p* ≤ 0.05

Most women were full-time housewives (79% vs. 92%; *p* < 0.001) in the intervention and control group respectively. However, more spouses of women in intervention group (62.7%) were engaged in non-farming jobs than those in the control group (24.6%) (*p* < 0.001).

### Utilization of healthcare services

#### Antenatal care

Table 2 shows the proportions of women who attended at least one antenatal care session in each trimester between the two groups. While about 36% of women in the control group attended ANC at least once in the 2nd and 3nd trimesters, 49% of respondents in the intervention group attended at least once (*p* < 0.01). Only 15% of women in the control group compared with 34% (*p* < 0.001) of women in the intervention group attended ANC at 36–38 weeks. For vaccination against tetanus, 62% of pregnant women in the control group compared to about 60% in the intervention group received tetanus toxoid vaccine (*p* = 0.52). More women in the intervention group (35%) received referral for HIV test than women in the control group (11%; *p* < 0.01).

**Table 2:** Health services uptake between intervention and control groups

Variables	Standard	Intervention	Chi-square test
	%(*n*)	%(*n*)	*p*-value
Antenatal care			
^b^Attended at least 1 antenatal care session in 2nd trimester	65.4(170)	55.6(143)	0.023
^b^Attended at least 1 antenatal care session in 3rd trimester	57.7(150)	89.1(229)	0.000
^b^Attended at least 1 antenatal care session in each (2nd & 3rd) trimester	36.2(94)	48.6(125)	0.004
^b^Attended ANC at 36-38wks	15.4(40)	33.9(87)	0.000
^b^Received Tetanus Toxoid during pregnancy	62.3(162)	59.5(153)	0.518
Postnatal care			
^c^Received OPV0 & BCG	24.2(62)	31.6(80)	0.063
^c^Immunization at 6 weeks	5.5(14)	14.2(36)	0.001
^c^Immunization at 10 weeks	4.3(11)	8.7(22)	0.044
^c^Immunization at 14 weeks	2.0(5)	6.7(17)	0.008
^c^Exclusive breastfeeding	2.3 (6)	1.4(4)	0.535
^b^Delivered at health facility	7.7(20)	12.8(33)	0.054
^c^Attended PNC within 2 weeks	28.5 (73)	32.8(83)	0.294
^c^Critical uptake of health services^a^	19.1(49)	29.3(74)	0.008

^a^Critical uptake defined as attending ANC in 3rd trimester, early postnatal care session (within 2 weeks after delivery) or immunization at birth

^b^(Control *N* = 260; Intervention *N* = 257)

^c^(Control *N* = 256; Intervention *N* = 253)

#### Delivery and postnatal care

Proportion of women who delivered at a health facility was significantly higher among women in the intervention group (13%) compared to those in the control group (8%) *p* < 0.001. Less than 10 % of respondents in both groups (intervention 1% vs. control 2%) exclusively breast fed their babies (*p* = 0.535). Immunization uptake for newborns differed between the control and the intervention groups. Overall, more neonates in the intervention group (32%) compared to those in the standard group (24%) *p* = 0.06 received *Bacillus Calmette–Guérin (*BCG) immunization and oral polio vaccine (OPV) at birth. This finding was similar for follow up immunizations at 6 weeks (14% vs. 6%; *p* = 0.001), 10 weeks (9% vs. 4%; *p* = 0.04) and 14 weeks (8% vs. 2%; *p* = 0.01). ‘Critical uptake of health services’ was significantly higher in the intervention group (29.3%) than the control group (19.1%; *p* = 0.01).

#### Effects of centering pregnancy on utilization of health services

Table 3 shows multivariate analyses of the effects of centering pregnancy on utilization of antenatal and postnatal services. When controlled for age, number of previous pregnancies, number of term deliveries, number of children alive and occupation of respondent, respondents who participated in the CPGs compared to those who did not, were more likely to attend at least one antenatal care (ANC) session in the third trimester [adjusted risk ratio (ARR):1.52; 95% CI:1.36–1.69]. They were also two times more likely to attend an ANC session between 36 and 38 weeks of gestation [ARR: 1.99; 95%CI: 1.40–2.84]. For uptake of immunization, babies born to mothers in the intervention group were more likely to receive immunization at six weeks [ARR: 2.23; 95% CI: 1.16–4.29] and fourteen weeks [ARR: 3.46; 95% CI: 1.19–10.01]. Critical uptake of health services (attending ANC in 3rd trimester and early postnatal care session (within 2 weeks after delivery) or immunization at birth) among women in the intervention group was 1.5 times more likely than in the control group [ARR: 1.50; 95% CI: 1.06–2.13].

**Table 3:** Multivariate analyses: effects of HMC+ on uptake of health services

Variables	Crude RR(95CI)	Adjusted^a^ RR(95CI)
Attended at least 1 antenatal care session in 2nd trimester		
Control	1.00	1.00
Intervention	0.85 (0.74, 0.98)	0.84 (0.73, 0.96)
Attended at least 1 antenatal care session in 3rd trimester		
Control	1.00	1.00
Intervention	1.54 (1.38, 1.73)	1.52 (1.36, 1.69)
Attended at least 1 antenatal care session in each (2nd & 3rd) trimester		
Control	1.00	1.00
Intervention	1.35 (1.10, 1.65)	1.10 (0.89, 1.37)
Received OPV0 & BCG^b^		
Control	1.00	1.00
Intervention	1.31 (0.98, 1.73)	1.32 (0.97, 1.81)
Immunization at 6 weeks		
Control	1.00	1.00
Intervention	2.60 (1.44, 4.70)	2.23 (1.16, 4.29)
Immunization at 10 weeks		
Control	1.00	1.00
Intervention	2.02 (1.00, 4.09)	1.98 (0.91, 4.32)
Immunization at 14 weeks		
Control	1.00	1.00
Intervention	3.44 (1.29, 9.18)	3.46 (1.19, 10.01)
Completed Immunization		
Control	1.00	1.00
Intervention	1.40 (0.70, 2.80)	1.19 (0.54, 2.58)
Exclusive breastfeeding		
Control	1.00	1.00
Intervention	0.67 (0.19, 2.36)	0.56 (0.14, 2.24)
Attended ANC at 36-38wks		
Control	1.00	1.00
Intervention	2.20 (1.58, 3.07)	1.99 (1.40, 2.84)
Received Tetanus Toxoid during pregnancy		
Control	1.00	1.00
Intervention	0.96 (0.83, 1.10)	0.92 (0.80, 1.06)
Delivered at health facility		
Control	1.00	1.00
Intervention	1.67 (0.98, 2.83)	1.09 (0.63, 1.90)
Attended PNC within 2 wks		
Control	1.00	1.00
Intervention	1.15 (0.89, 1.50)	1.18 (0.89, 1.58)
Critical uptake of health services		
Control	1.00	1.00
Intervention	1.53 (1.11, 2.10)	1.50 (1.06, 2.13)

^a^Each model controls for extraneous effect of respondents’ age, number of previous pregnancies, number of term deliveries and number of children alive on each outcome indicator

^b^Bacillus Calmette–Guérin

## Discussion

This is the first study in Nigeria and to the best of our knowledge sub-Saharan Africa that has assessed the effects of centering pregnancy on utilization of maternal and new-born healthcare services and we identified several important observations. First, implementing the centering pregnancy approach is feasible in rural settings with limited health workers. Secondly, women in the CPG showed higher utilization of antenatal and delivery services. Thirdly, infants born to women in the CPG were more likely to receive complete dose of immunization with reference to the schedule of immunization at birth, 6, 10 and 14 weeks. Lastly, “critical uptake of healthcare” - attending ANC at 36 weeks, having an early postnatal care visit (within 2 weeks) and a new-born receiving immunization at birth was more likely among women who participated in the CPG. These findings have salient implications for reduction of maternal mortality in Nigeria, maternal health programs, policy and funding.

The World Health Organization recognizes the practice of Centering Pregnancy, but does not recommend it as a routine intervention but for research [25]. Studies on Centering Pregnancy are few and most have been in developed countries. A pilot study in Malawi demonstrated the feasibility of implementing group ANC by assessing adherence to thirteen essential elements of CP, however no clinical outcomes were measured [26]. Centering Pregnancy has been shown to improve perinatal outcomes including prenatal care and knowledge, and decreased HIV risk [27–29]. One of the earliest studies on CP found high rates of ANC utilization (86%), low rates of preterm delivery (4.5%) and low birth rates (5.4%), and fewer emergency visits in the third trimester [16, 30]. In a prospective matched cohort design, birth weight for preterm infants were significantly higher among women in CPG compared to standard of care (2398 g vs. 1990 g; *p* < 0.05) [16, 27]. Positive effects of CP have also been demonstrated among adolescents. Grady and Bloom (2004) found significantly lower preterm (11% vs. 26%; *p* < 0.02) and low birth weight (9% vs. 23%; *p* < 0.02) rates and higher satisfaction among young women who had enrolled in CPGs [28]. A randomized controlled trial of CP in the United States of America, found that women in the CPG had a 33% reduction in preterm birth as well as significant improvements in breastfeeding initiation and maternal prenatal knowledge [16, 28]. Klima et al. [16] also demonstrated better perinatal outcomes including more ANC visits among women enrolled in CP in their study in United States. In our study, respondents in the CPG were more likely to attend ANC in the third trimester and at late stages of pregnancy (36–38 weeks) which indicates that with closer monitoring provided by CPG, utilization of essential services can be increased. However, the overall low utilization of antenatal care services observed in both intervention and control groups in this study is similar to findings reported in other studies conducted in the North-West region of Nigeria. ANC utilization has been consistently low in north west Nigeria (36–41%) than the national average of 50% in 2012 and 2013 [13, 14]. Thus, given the regional low utilization of ANC services, evidence-based programs that demonstrate the potential for increased utilization must be promoted.

Most women enrolled in this study delivered outside the health facility setting. This is similar to regional estimates for North West Nigeria, with home deliveries averaging about two-thirds of deliveries in 2012 and 2013 [14]. In the Northwest region of Nigeria, vast majority of deliveries (90.3%) still occur at home, with 67% of women not receiving ANC during their last pregnancy. Furthermore, 37% of women in north west Nigeria and 63% in Kano state reported that facility-based delivery was not necessary [14]. According to the Nigeria Demographic and Health Survey (NDHS, 2013), percentage of deliveries in a health facility in Kano was reported as 12% compared with the national average of 36%. Our study showed that more women in the CPG delivered in health facilities than those in standard care. However, this finding was not significant at the multivariate level which is probably a reflection of the general lack of trust in the health system and the strong influence of socio-cultural practices and beliefs related to childbirth. Socio-cultural beliefs have been reported to be key factors associated with uptake of maternal and newborn services as well as infant and under-five mortality. Societies are inherently imprisoned by their culture and history and the roots of contemporary health successes lie far back in those histories [31]. Several studies have reported the non-association of ANC utilization and facility-based delivery [32–38]. Berhan and Berhan (2014) in a systematic review of the role of ANC on facility-based delivery, found wide variations in twenty-two African countries between ANC utilization and facility-based delivery [38]. They reported that increase in health facility delivery among pregnant women attending antenatal care in their analysis was probably because of their awareness of the benefits, advantage or their familiarity with health facility environments and health care providers where they have been attending [35, 36] Another reason may be due to the ANC creating an informal forum to discuss and share information about pregnant women who were identified as ‘being at higher risk’ but ended up with uneventful deliveries [38, 39] in health facilities and combined with their uneventful ANC history, the socio-cultural influence and practice of home delivery takes precedence over facility-based delivery. A direct involvement of the family members and communities where pregnant women live may be a critical component of the intervention to improve outcomes. Families and communities often consider pregnancy as a natural process of life and therefore, underestimate the importance of ANC and facility-based delivery [8, 40]. Research has shown that programs that involve community members in developing, implementing, and monitoring are more likely to be acceptable to the community as well as have more effective outcomes. Conversely, failure to involve the community may not only result in failed intervention, but may also produce unforeseen and possibly adverse effects. Community involvement affects norms and contextual factors to create an environment favorable to changes in behavior that may decrease the vulnerability of individuals and groups at risk within the community [41]. Nevertheless, further qualitative research is urgently required to understand the reasons why home delivery is still preferred to facility-based delivery despite being exposed to the health facility.

Immunization against childhood diseases remains an effective public health intervention for reducing the burden of diseases attributable to tetanus, tuberculosis, polio, pertussis, diphtheria and hepatitis B. However, none of the few studies on CP reported an association between CP and the postnatal care, specifically immunization uptake. Thus, our study presents new findings for the scientific literature. Though the overall proportion of infants immunized was low, proportions of babies immunized at six, ten and fourteen weeks, were comparatively higher among infants born to women in the CPG. The low immunization uptake is consistent with regional estimates in 2013 which showed a decrease in immunization rates of 18 to 22% and 14 to 18% between the second and first and between the second and third schedules respectively for pertussis, diphtheria and tetanus [14]. Among the four schedules of immunization, immunization at birth was the highest at 32% and lowest at fourteen weeks (7%) among infants from the CPG. This finding was similar for the control group. However, the difference between the groups was not significant for immunization at birth (32% vs. 24%; *p* = 0.063). Given the low level of facility-based delivery, this finding is expected. However, for other schedules of immunization, it was significantly higher among infants whose mothers enrolled in the CPG. A plausible explanation for this may be due to home visitation and follow-up postnatally of women who enrolled in the CPG. This activity enabled health workers, identify those who had delivered at home and reinforced the benefits of immunization to these women. When controlled for confounders, infants born to women enrolled in CPG were twice as likely to receive immunization at six weeks and four times more likely at fourteen weeks. This suggests that CP has the potential to retain mother-baby pairs within the health system and allow close monitoring in early infancy and improving perinatal outcomes. Maternal uptake of tetanus toxoid was low and similar between groups which merits discussion. Only 60% of women in both groups received at least one dose of tetanus toxoid despite 89% of women in the intervention group attending ANC in the third trimester. This similarity may reflect the knowledge of the dosing schedule of tetanus toxoid for women and thus requires further investigation. The implication of this is that, 40% of infants have no passive protection against tetanus through transplacental transfer and more alarming is that this proportion may be lower if the assessment was based on women receiving the complete schedule. Studies have shown that receiving only one dose of tetanus toxoid confers no protection against tetanus [42]. Furthermore, with less than 15% of infants receiving DPT vaccine at 6 weeks post delivery, there’s an increased risk of neonatal tetanus. However, given that only a quarter of the women were primigravidas (first time being pregnant), it’s more likely that they’ve been previously exposed to the vaccine and thus will confer some passive protection to their infants.

Lastly, prevalence of exclusive breastfeeding was very low in this study and there was no difference between groups. Though this finding is alarming, it’s not surprising. Only 10% of infants in Nigeria are exclusively breastfed for up to 4–5 months [14]. More worrisome is that the median duration of exclusive breastfeeding is 0.5 months which indicates that 50% of infants in Nigeria are not exclusively breastfed for up to one month. Klima et al. [16] showed that women enrolled in CP were significantly more likely to have initiated at least some breastfeeding at hospital discharge and a higher proportion were exclusively breastfeeding at discharge compared to women in individual care (44% vs. 31%; *p* < 0.05). A similar study in Ghana [43], showed that more women in the CPG group were aware to start breastfeeding as soon as possible after birth and to exclusively breastfeed for at least six months (90% vs. 76%; *p* = < 0.01). More research is required to understand the reasons for very low exclusive breastfeeding rates.

This study has some limitations. The quality of antenatal care, shown to be one of the determining factors for women going to health facilities during labor [32, 33, 36, 38, 43–45] was not assessed in this study. Another limitation was the lack of randomization, thus we cannot rule out the possibility that women in the CPG were inherently more committed to ANC visits, more likely to engage in healthier behaviours and more likely to have a critical uptake of health services. However, given the similarities in socio-cultural backgrounds, it’s likely that the differences observed are attributable to CPG. The use of only one local government area in Kano state also limits our ability to generalize our findings. Lastly other factors such as inability to pay for ANC services, distance to health facility, long wait times and other socio-demographic factors which have been shown to be associated with utilization of ANC services were also not assessed in this study.

## Conclusion

In conclusion, this is the first study to assess the effects of CP on maternal and infant health care utilization and our findings indicate that CP is a promising approach to increase use of health facilities for both mother and child. The increased utilization of ANC services among women in the CPG allows the increased exposure of pregnant women to health care providers and consequently the early identification of high-risk pregnancies and early referral to higher levels of care. This has a direct impact on reducing adverse events including maternal mortality. The higher immunization rates among infants of women enrolled in the CPG is commendable and will add the necessary boost to improving the national immunization rates for the childhood killer diseases. Lastly, the similarity in facility-based delivery observed between intervention and control group despite increased likelihood of a third trimester visit by women in the CPG suggests that ANC utilization has a relative advantage but is not a solution by itself for facility-based delivery. In other words, antenatal care is a necessary intervention but not a sufficient factor in predicting the probability of birth in health facility [38]. However, given the higher facility-based delivery observed, CP should be funded and promoted in communities with poor facility-based delivery rates. The synergy that will be achieved from this will be pertinent to reducing the high maternal mortality rate in Nigeria.
